# Management of chronic myeloid leukemia in 2023 – common ground and common sense

**DOI:** 10.1038/s41408-023-00823-9

**Published:** 2023-04-24

**Authors:** Jayastu Senapati, Koji Sasaki, Ghayas C. Issa, Jeffrey H. Lipton, Jerald P. Radich, Elias Jabbour, Hagop M. Kantarjian

**Affiliations:** 1 Department of Leukemia, The University of Texas MD Anderson Cancer Center, Houston, TX USA; 2 Cancer Clinical Research Unit, Princess Margaret Cancer Centre, Toronto, ON Canada; 3 Clinical Research Division, Fred Hutchinson Cancer Center, Seattle, WA USA

**Keywords:** Disease prevention, Health services

## Abstract

With the improving knowledge of CML and its management, the goals of therapy need to be revisited to ensure an optimal use of the BCR::ABL1 TKIs in the frontline and later-line therapy of CML. In the frontline therapy of CML in the chronic phase (CML-CP), imatinib and the three second-generation TKIs (bosutinib, dasatinib and nilotinib) are associated with comparable survival results. The second-generation TKIs may produce earlier deep molecular responses, hence reducing the time to reaching a treatment-free remission (TFR). The choice of the second-generation TKI versus imatinib in frontline therapy is based on the treatment aims (survival, TFR), the CML risk, the drug cost, and the toxicity profile with respect to the patient’s comorbidities. The TKI dosing is more flexible than has been described in the registration trials, and dose adjustments can be considered both in the frontline and later-line settings (e.g., dasatinib 50 mg frontline therapy; dose adjusted schedules of bosutinib and ponatinib), as well as during an ongoing TKI therapy to manage toxicities, before considering changing the TKI. In patients who are not candidates for TFR, *BCR::ABL1* (International Scale) transcripts levels <1% are acceptable, result in virtually similar survival as with deeper molecular remissions, and need not warrant a change of TKI. For patients with true resistance to second-generation TKIs or with the T315I gatekeeper mutation, the third-generation TKIs are preferred. Ponatinib should be considered first because of the cumulative experience and results in the CML subsets, including in T315I-mutated CML. A response-based dosing of ponatinib is safe and leads to high TKI compliance. Asciminib is a third-generation TKI with possibly a better toxicity profile, but lesser activity in T315I-mutated CML. Olverembatinib is another potent third-generation TKI with early promising results.

## Introduction

Today, we celebrate two decades of experience with imatinib and later-generation BCR::ABL1 tyrosine kinase inhibitors (TKIs) in Philadelphia chromosome (Ph)-positive chronic myeloid leukemia (CML). With maturing data, it is important to review our established guidelines on treatment options in frontline and subsequent-line CML therapy, treatment aims, response monitoring, and the significance of the response milestones proposed by the European LeukemiaNet (ELN) and the National Comprehensive Cancer Network (NCCN) [1–6]. In this review, we discuss these issues to clarify questions about the management of CML in frontline and post-frontline TKI failure settings.

## Frontline CML therapy

The primary aim of CML therapy is to improve survival so that it matches that of a normal population. A second aim, emphasized in the past decade but that benefits fewer patients, is the achievement of a durable deep molecular response (DMR), which can then allow treatment discontinuation and potentially a treatment-free remission (TFR) status [7].

Four BCR::ABL1 TKIs are approved for frontline therapy: imatinib, dasatinib, bosutinib and nilotinib. All four produce near-normal quality of life and life expectancy provided patients comply with the treatment, are monitored optimally with minimal interruptions in therapy, and are managed appropriately at the earliest sign of true treatment resistance [8–13]. Imatinib is available as a generic drug in the United States (US), and at least one of the 15 available formulations is priced at about $500/year in the US (through Cost Plus Drugs, for example) [14–17]. Imatinib generics are routinely available for $300-$3,000/year in other regions. Dasatinib will be available as a generic formulation in the US by 2024 and can be prescribed elsewhere now. The prices of patented TKIs range from $150,000 to 250,000+/year [18, 19]. Therefore, an important consideration in the frontline CML therapy is the “treatment value” or cost-benefit of a TKI if overall survival (OS) is the treatment endpoint. For this aim, generics provide the best treatment value [17, 20, 21].

The choice of frontline therapy may depend on additional factors:

- Aim of therapy (survival or TFR): Among older patients, survival may be the primary aim, and TFR secondary. Among such patients, imatinib may be the better frontline TKI therapy. In younger patients, TFR may be pursued more aggressively by some CML experts and patients, with changes in therapy in the absence of a DMR (*BCR::ABL1* transcripts on International Scale [IS] ≤ 0.01%) after 3–5 + years of frontline TKI therapy (discussed later).
- Cost of the TKI and affordability to the patient: In the US, this may be tied to the out-of-pocket expenses, which can be as high as 25% of the price [20, 22, 23]. In such instances, generic TKIs may be the only realistically affordable option [16, 17, 19].
- Patient co-morbidities: Some notable co-morbidities that influence the choice of a TKI include chronic lung disease, hypertension, diabetes, hepatic or renal dysfunction, pancreatitis, enterocolitis, vaso-spastic or occlusive events, and others. These will be discussed in details under “ Management of CML post TKI toxicities”.) [24].
- CML risk category: In patients with higher-risk disease (as defined by the Sokal or other risk models), the second-generation TKIs may be favored over imatinib by some CML experts and in community practice as frontline therapy [25–28]. No advantage has been observed in lower-risk disease. Recent studies have suggested the possible adverse effects of molecular abnormalities like *ASXL1* mutations, but these are not yet incorporated into the CML risk models.

## What is the true incidence of resistance to optimal frontline TKI therapy?

It is often stated that the incidence of primary resistance to frontline TKI therapy is 10%, and of secondary resistance 30% [29]. This may have been true in the original TKI studies, with the suboptimal use of imatinib, and may depend on the definition of resistance. True resistance to frontline therapy may be significantly lower. In the German CML IV trial of 1551 patients treated in chronic phase (CP) with imatinib-based regimens, with a median follow-up time of 10 years, the 10-year OS rate was 82%, the relative survival rate 92%, and the cumulative incidence of blast phase (BP) only 5.8%. Only 26.5% of patients switched from imatinib to second-generation TKIs, 10% because of resistance, the others for toxicities and/or other reasons [10]. In our experience with frontline lower-dose dasatinib therapy (50 mg daily), with a median follow-up of 5 years, the incidence of true primary plus secondary resistance (defined as *BCR::ABL1* transcripts [IS] > 1% any time after 12 months of therapy) was < 5%. The estimated 5-year OS rate was 98%, with only two deaths (not related to CML) and no transformation events to accelerated phase (AP) or BP [30, 31]. Similar results were reported in the randomized trial of dasatinib 100 mg daily versus imatinib in frontline CML therapy [32]. In our long-term frontline TKI therapy experience, the estimated 15-year OS rate (including any death, regardless of cause) is about 75%. The CML-specific survival rate (considering only deaths from CML or treatment complications) is >90% [33]. Thus, frontline therapy with the existing TKIs achieves the primary endpoint of survival normalization for most patients with CML.

## How about the achievement of TFR?

Here, we discuss the definition of DMR and its optimal duration before considering TKI discontinuation. It is increasingly accepted that a DMR does not necessarily require negative results for *BCR::ABL1* transcripts but could include levels that represent a reduction of 4 (MR4) or 4.5 logs (MR4.5). Discontinuing the TKI after a sustained DMR of >2 years results in 3-year TFR rates of 40%–50% [7, 34–37], while discontinuation after a DMR of ≥5 years results in a 5-year TFR rate of >80% [38]. Hence, in the absence of severe toxicities, more CML experts and patients are leaning toward continuing the TKI therapy for longer durations before considering stopping. The achievement of TFR has been estimated to be about 25–30% [34–36, 39]. This may be an underestimation of the true TFR rate. Assuming that 70%–90% of patients achieve a DMR with imatinib or second-generation TKIs, which is durable in about 80%, and also assuming that discontinuation occurs after 5 or more years of DMR, then the TFR rates should be 40%–55% of the total population treated. This assumes that all eligible patients are willing to discontinue TKI therapy, which they are often reluctant to do.

An important question that cannot be answered with objective data is the likelihood that a patient who is on imatinib or a second-generation TKI for >5 years and who has not achieved a DMR will be able to reach the goal of TFR by changing TKI therapy? The assumption that it is possible to increase the TFR rate by rotating the second-generation TKIs or changing to a third-generation option has been the rationale by many physicians (also encouraged by CML experts, and in CML reviews, advisory boards and symposia) to change the TKI in a patient who does not achieve a major molecular response (MMR; *BCR::ABL1* transcripts [IS] < 0.1%) or DMR after some duration of TKI therapy. While common, this practice may not be successful and is the subject of recent discussions among CML experts.

## How important is it to achieve the landmark optimal responses recommended by the ELN and NCCN?

The ELN recommendations and NCCN guidelines highlight the importance of achieving an “early molecular response” (EMR; defined as *BCR::ABL1* transcripts [IS] < 10% at 3–6 months) and a complete cytogenetic response (CCyR; approximated to *BCR::ABL1* transcripts [IS] < 1 %) by 12 months of therapy [5, 6].. In the early years of the TKIs, EMR was emphasized, resulting in attempts to optimize therapy by switching from imatinib to second-generation TKIs in the first few months of therapy [40, 41]. In the German CML IV experience, achieving *BCR::ABL1* (IS) transcripts < 10% at 3 months was associated with a 5-year OS rate of 94%, compared with 90% if levels were >10% [10]. Indications from recent experiences are that the 6-month timepoint is more important than the 3-month timepoint for EMR, and that lack of EMR at 6 months occurs in a minority of optimally treated and compliant patients (< 5% with second-generation TKIs) [41–43].

Historical experience with interferon (IFN) alpha showed that the achievement of a major cytogenetic response (roughly equivalent to *BCR::ABL1* transcripts [IS] < 10%) or CCyR was associated with excellent long-term survival [44]. However, once imatinib and other highly effective TKIs became available, enthusiasm was high and the prevailing practice became to achieve the deepest responses possible (even when not aiming for TFR), regardless of the costs related to frequent changes of TKIs and their potential additional toxicities. Earlier and deeper molecular responses were assumed to be reliable surrogate endpoints for long-term survival. This did not turn to be the case, perhaps because of the availability of highly active salvage therapies for patients who progressed or lost a CCyR after >1 year of frontline TKI therapy. More recently, some studies have re-analyzed the associations between outcome and responses less than MMR. Shaya and colleagues reported that patients who did not achieve a CCyR after 2 years of TKI therapy had a significantly worse survival than those who did [45]. The estimated 10-year survival rates were 75% versus 90% (HR 0.36; *p* < 0.001). This was statistically and clinically significant, but showed than even patients who did not achieve a CCyR did reasonably well. The investigators did not separate the results based on transcript levels < 10% versus > 10%. In a second study of 131 patients not achieving MMR after 2 years of TKI therapy, Bidikian and colleagues observed that the 10-year CML-specific survival rate was similar (95%) among patients with *BCR::ABL1* transcripts (IS) > 0.1%–1% and >1%–10% [46]. Only patients with levels > 10% at 2 years had a worse 10-year OS rate, at 80%. Thus, as with the IFN alpha experience, achieving *BCR::ABL1* transcripts (IS) < 10% (≈MCyR) translated into a reasonable long-term survival. This finding, which needs to be confirmed in other studies, may be more important in the management of older patients in whom allogeneic stem cell transplantation (SCT) is considered when the *BCR::ABL1* (IS) transcripts persist at levels of 1%–10%.

One of the most common questions posed in CML practice is how to treat a patient of a particular age (from 15 to 90 years old) on frontline therapy with a particular TKI (e.g. imatinib, dasatinib, bosutinib, or nilotinib) and in whom the *BCR::ABL1* transcripts (IS) are anywhere from 0.01 % to 0.5%. A frequent reaction is to rotate second-generation TKIs or use a third-generation option (ponatinib, asciminib). This is driven by the emphasis in the past two decades on the paramount importance of achieving CCyR (≈ *BCR::ABL1* transcripts <1%), MMR, or DMR in order to pursue a TFR. Being too aggressive in pursuing these goals may result in harmful effects, including new TKI toxicities, financial burdens and added stress for the patient [47]. Once a patient is determined to have a low probability of a TFR (i.e. no durable DMR after >5 years of TKI therapy), changing TKIs may cause more harm than potential benefit. Another course of action—which may be different from other CML experts’ opinions —would be to continue with the same TKI and monitor the patient’s *BCR::ABL1* transcripts at frequent intervals (every 3 months if transcripts >0.1%; every 6 months if ≤0.1%).

## Management of CML post frontline TKI therapy failure

Despite the efficacy of frontline TKI therapy in CML, treatment failure occurs in a minority of patients who then require salvage therapy with other TKIs, allogeneic SCT, non-TKI therapies (omacetaxine mepesuccinate, cytarabine, hypomethylating agents, hydroxyurea, venetoclax), or combinations.

Failure to frontline therapy can be due to 1) TKI toxicities or 2) treatment resistance [48, 49]. Poor compliance to therapy can be a major contributor to treatment failure, and can be caused by inadequately managed drug toxicities, financial burdens and other causes [50, 51]. Historically, the incidence of failure was quoted to be about 50%, with studies showing that after 5 years of frontline TKI therapy, about 40%–60% of patients were on alternative TKIs [52, 53]. This information was used to emphasize the need to develop and approve more and better TKIs, which was reasonable.

As knowledge of outcomes and experiences is gained, it appears that, in the early period of TKIs in CML, patients on a frontline TKI were often changed to other TKIs for indications that are not considered as often today. For example, many patients were advised to change from imatinib or a second-generation TKI to a different second-generation option when the *BCR::ABL1* (IS) transcripts were > 10% at 3 months, > 1% after 1 year, or even 0.1% to < 1% after 1 to ≥ 5 years of therapy. Also, patients are at times advised to change TKIs even for *BCR::ABL1* transcripts (IS) < 0.1%, in order to pursue TFR. In addition, when patients experienced toxicities on a TKI, they were often changed to another, regardless of the nature and severity of the toxicity, rather than attempting to lower the dose, if the symptoms were reversible or mild-moderate, and when patients were already in an acceptable state of molecular remission [54]. This was perhaps the reason for the higher rates of reported “treatment failure” than seen today. At MD Anderson, using dose reductions for toxicities and judicious changes of therapy only for clinically significant changes in molecular response, >80% of patients remain on the frontline TKI after 5 years of therapy.

## Management of CML post-TKI toxicities

Some toxicities are highly TKI-specific **(**Table 1**)** [55]. Common side-effects with imatinib therapy include fluid retention, periorbital edema, bone and muscle aches, and, less commonly, weight gain. Rare events, include renal dysfunction and neurotoxicity (dementia-like; parkinsonism) [11]. Dasatinib is associated with pleural effusions, and myelosuppression; rarely, patients develop pulmonary hypertension and muscle aches [32, 56]. Bosutinib is associated with gastrointestinal (GI) toxicity (diarrhea), and hepatic and renal dysfunction [57, 58]. Nilotinib can exacerbate hyperglycemia and cause dyslipidemia [59, 60]. After a 10-year follow-up, the incidence of arterio-occlusive events(AOEs) and veno-occlusive events (VOEs) (angina, myocardial infarction, cerebrovascular accidents, transient ischemic cerebral events, peripheral arterial insufficiency) was 24.8% with nilotinib 300 mg BID and 33.4% with nilotinib 400 mg BID [24]. Imatinib and nilotinib can rarely cause pancreatitis [24, 59, 61, 62]. Ponatinib is probably the most effective but more toxic TKI when used at a dose of 45 mg daily [63–65]. It is associated with systemic hypertension (> 20%), AOEs (10–20%), skin rash (10–20%) and pancreatitis (5%) [65–68].

**Table 1:** Clinically relevant TKI toxicities and reduced dose schedules.

TKI	Common side effects	Toxicities to watch for	^a^Prohibitive toxicities	^b^Lowest dose range
**Imatinib**	Rash, fluid retention, edema, weight gain, musculoskeletal aches, diarrhea, skin depigmentation	Renal toxicity	Neurotoxicity	100–200 mg/day
**Nilotinib**	Rash, headaches, increased bilirubin, impaired glycemic control, dyslipidemia	Renal toxicity, pancreatitis, Worsening diabetes	Arterio-occlusive and vaso-occlusive events	200 mg/day–200 mg BID
**Dasatinib**	Pleural effusion, cytopenia	Pulmonary hypertension, systemic hypertension	>1 episode of pleural effusion, pulmonary hypertension	20–50 mg/day
^c^**Bosutinib**	Gastrointestinal toxicity (diarrhea/colitis), renal dysfunction, liver dysfunction	Enterocolitis	Enterocolitis	100–200 mg/day
**Ponatinib**	Rash, hypertension	Pancreatitis, hepatic toxicity	Arterio-occlusive and vaso-occlusive events; refractory hypertension	15 mg/day

*TKI* tyrosine kinase inhibitor, *BID* twice daily.

^a^Clinical pancreatitis is a prohibitive toxicity that can occur with all TKIs, though most common with nilotinib and ponatinib.

^b^Lowest dose-range is a dynamic therapy decision that depends on the burden of the symptoms, any patient comorbidity that could be additive to the toxicity, and the state of CML disease control.

^c^For bosutinib a slow dose escalation over 3–4 months (100 mg/day x 1–2 weeks, 200 mg/day x 2–4 weeks, 300 mg/day x 1 month) to reach the final dose of 400 mg/day or 500 mg/day might ameliorate the gastrointestinal toxicities.

Knowing the common side effects of a TKI can help in the selection of the drug based on patient co-morbidities, as discussed earlier (under the choice of frontline therapy) [69]. While we once assumed that these toxicities were particular to certain TKIs, recent data showed a higher-than-expected rate of TKI cross-intolerances [55, 68, 70, 71]. In a registry analysis from Canada, the reason for treatment failure post frontline TKI therapy was about 57% due to intolerance and 43% due to resistance. However, with subsequent failures, intolerance became a more common cause of treatment failure and often recurred in the same patients [72].

### Value of TKIs at lower dose schedules

TKI toxicities are strongly associated with the higher dose schedules. Originally, the TKIs (and many of the novel recent targeted therapies) were developed in strategies similar to the ones used for chemotherapy, at one dose lower than the maximum tolerated dose (MTD), which was based on the first 1-2 courses of therapy [73]. However, chemotherapy regimens were used for short durations of 6–12 months. In contrast, some of the targeted therapies are needed for years, and, at times, for the patient’s lifetime. This has uncovered late toxicities not observed with the shorter follow-ups (e.g. AOEs, pleural effusions, and organ dysfunctions with CML TKIs). It was also observed that efficacy could be similar, or maintained after a response is achieved, at lower dose schedules [30, 54, 74–76]. Thus, original experiences with the TKIs in CML highlighted a new concept in cancer therapy: developing such long-term targeted therapies at an “optimal biologic dose” (OBD), rather than at the lower-than-MTD dose [65, 77–80].

Thus, over the past decade, we have learned that dasatinib 50 mg daily is safer and as effective as 100 mg daily [30, 31, 81]. Bosutinib side-effects can be mitigated with a dose-escalation schedule: 100 mg daily x 1-2 weeks, then 200 mg daily x 2–4 weeks, then 300 mg daily x 1 month, then adjust the dose to 400 mg daily (approved dose in frontline therapy) or a lower or higher dose (500 mg daily; approved in subsequent-line therapies) depending on the response, CML status (frontline or later lines) and side-effects [82, 83]. Nilotinib can be de-escalated safely from 300–400 mg BID to 150–200 mg BID, or even 200 mg daily, if side-effects occur, or if there are safety concerns in patients who have responded optimally [84, 85]. Recent studies have also shown that dose-adjusted ponatinib schedules (e.g. starting at 45 mg daily in T315I mutated CML, 30 mg in others, and reducing the dose to 15 mg daily once *BCR::ABL1* transcripts [IS] are <1%) are as effective and significantly safer than a fixed dose of 45 mg daily (reduced only if toxicities) [65, 67, 80].

Asciminib was approved in 2021 for the third-line therapy of CML and for the treatment of T315I-mutated CML. Emerging experiences suggest its safety and efficacy with the short-term follow-up [86–89]. Though all grade and ≥ grade 3 AEs were lower with asciminib than bosutinib in the ASCEMBL trial, when studied at the higher 200 mg BID dose for T315I-mutated CML, asciminib showed ≥ grade 3 adverse events (AEs) in 60% of patients and AOEs in 8% of patients [87, 90]. However, longer follow-up in larger patient numbers will define better its safety [91].

Based on the above clarifications, our recommendations are as follows: For a patient on frontline imatinib therapy, if toxicities emerge in the setting of a good molecular response, the imatinib dose can be reduced to 300 mg or 200 mg daily before considering a TKI change. Response by peripheral blood *BCR:ABL1* testing should be carefully monitored after dose reduction. Alternatively, a second-generation TKI can be swapped for imatinib. However, if the patient is already responding well on imatinib, then the dose of the second-generation TKI may not have to be the salvage approved dose (dasatinib 100 mg daily, nilotinib 400 mg BID, bosutinib 500 mg daily). Rather, the lower-dose schedule can be used more safely and with equal or better efficacy (dasatinib 50 mg or even 20 mg daily; nilotinib 150–300 mg BID or 200 mg daily; bosutinib 200–300 mg daily). Again, this strategy should be safe in the context of closely following the disease burden by *BCR::ABL1* transcripts monitoring. If the patient has CML failure on second-line TKI therapy because of similar or new toxicities, the second-generation TKIs can be rotated (dasatinib, bosutinib, nilotinib) based on the type of toxicity and patient’s comorbidities. If toxicities occur with all the four TKIs after dose adjustments, then a third-generation TKI can be used (for example, ponatinib, not 45 mg daily, but rather 15 mg daily since most of these patients would have *BCR::ABL1* transcripts (IS) < 1%). Asciminib 40 mg BID or 80 mg daily would be another third-generation TKI option. In a situation where patients start with second-generation TKIs as frontline therapy (more common today) and have toxicities with other second-generation TKIs, imatinib can be an excellent TKI option with low side effects and low cost.

While most TKI toxicities resolve with dose reductions, there are some for which this strategy might not be appropriate: 1) recurrent pleural effusions (if more than once with dasatinib after dose reductions; less commonly with other TKIs such as bosutinib); 2) pulmonary hypertension (usually on dasatinib therapy; unlike the common belief, in some patients it can resolve over time with a short course of steroids and sildenafil) [92, 93]; 3) VOE or AOE with ponatinib, nilotinib or other TKIs (bosutinib and imatinib are the safer TKIs in such instances); 4) enterocolitis with bosutinib (and less often with dasatinib; rarely patients on bosutinib with enterocolitis have undergone unnecessary bowel resections because bosutinib was not held) [58]; 5) dementia conditions, including, rarely, Lewy body-like dementia, or parkinsonism (can resolve weeks to months after discontinuing the TKI); 6) immune-mediated myocarditis, hepatitis or nephritis. In the above situations, changing to another TKI rather than dose reduction would be the safer choice. Table 1 summarizes the common toxicities with the TKIs and the recommended dose reductions.

## Management of CML post-TKI resistance

In this section, we discuss the therapy of resistant CML based on *BCR::ABL1* transcripts (IS) > 1% after more than one year of frontline TKI therapy, or after adequate second-line therapy at an optimal TKI dose schedule given for 3–6 months.

Among patients who develop resistance to frontline imatinib therapy, changing to a second-generation TKI is the most appropriate course. The choice of the TKI depends on the patient’s comorbidities and on the *ABL1* kinase domain (KD) mutations, which should be performed on all patients with TKI resistance considered for a change of therapy (can be guiding in 50%) [94]. More than 100 *ABL1* KD mutations have been reported; on asciminib therapy, new mutations involving the myristoyl pocket (site of asciminib binding) are emerging [95]. Table 2 shows the in-vitro sensitivity profiles of the TKIs to different *ABL1* KD mutations. Patients who develop resistance on frontline or subsequent-line TKIs (dasatinib, bosutinib, nilotinib), should not be rotated to other second-generation TKIs unless indicated by a guiding mutation. In such patients, changing to a third-generation TKI (ponatinib, asciminib) is appropriate [96].

**Table 2:**
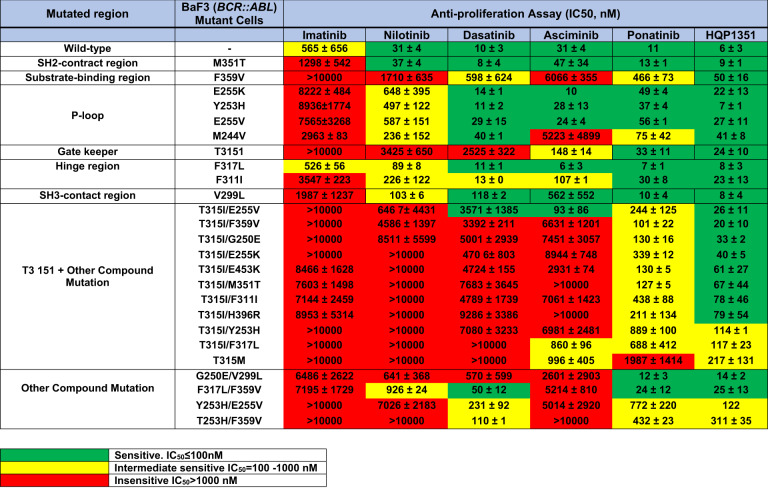
The spectrum of TKI sensitivity according to the different ABL1 kinase domain mutations, including compound mutations in Ba/F3 cells.

(Data provided by Ascentage pharma)

An alternative for younger patients is allogeneic stem cell transplantation (SCT), which remains highly curative as a one-time procedure [97–99]. With the choices of stem cell donor increasing (matched sibling, matched unrelated, haplo-identical, and umbilical cord), SCT is now an option for most patients if needed. In older patients, it is perhaps reasonable to forgo allogeneic SCT, with its potential morbidity, in favor of strategies that can maintain patients in CML-CP (albeit not in CCyR) for a decade or more. These include combining the most optimal TKI with other agents such as hypomethylating agents (decitabine, azacitidine), hydroxyurea, low-dose cytarabine, or omacetaxine mepesuccinate (though for 2–5 days/month rather than the approved two weeks/month schedule, which can be very myelosuppressive) [100–102]. In such instances, maintaining good disease control with a partial or even no cytogenetic response might be acceptable. The key to optimal therapy is to maintain a daily TKI dose schedule. As discussed above, the 10-year CML-specific survival could be as high as 90% among patient with *BCR::ABL1* transcripts (IS) 1–10%, and 75% with transcripts >10% [46]. Because of its different mechanism of action, asciminib is being combined with other TKIs in ongoing investigational trials. This approach should not be carried into the standard practice since such combinations may increase the cost of care significantly and may be associated with unforeseeable longer-term synergistic toxicities.

## Third-generation TKIs

The development of resistance in a subset of patients on second-generation TKIs, and of T315I mutations (resistant to imatinib and all second-generation TKIs) provided a therapeutic niche for third-generation TKIs.

### Ponatinib

The first third-generation TKI to receive regulatory approval was ponatinib, which was specifically designed to bypass the *ABL1-*T315I KD gatekeeper mutation [103, 104]. In in-vitro studies, ponatinib had a dose-dependent inhibition of the Abl kinase activity with the highest doses required for cells with *ABL1*-T315I, E255V mutations and compound mutations **(**Table 2**)** [104]. Ponatinib was originally developed at a dose of 45 mg daily, based on the Phase 1 study establishing it as the Phase 2 dose [63, 64, 105]. However, recent studies showed that lower dose schedules were effective and safer [65, 67].

The Phase 2 PACE trial accrued 449 patients, including 267 patients with CML-CP (203 patients with prior TKI resistance or intolerance; 64 patients with T315I mutation), 145 patients with advanced phase CML (83 with AP; 62 with BP), and 32 patients with Ph-positive acute lymphoblastic leukemia (ALL) who received ponatinib 45 mg daily [63]. Among patients with CML, > 90% had received ≥2 prior TKIs, and 60% had received ≥3 prior TKIs. In CML-CP, among patients with prior TKI resistance or intolerance, 50% achieved CCyR and 35% an MMR. Among patients in CML-CP with a T315I mutation, 68% achieved CCyR and 56% had MMR. Eight of 14 (57%) CML-CP patients who had a compound *ABL1* KD mutation that included T315I attained a CCyR. In the second, third and ≥ fourth line TKI settings in CML-CP, the CCyR rates were 74%, 56%, and 38%, and MMR rates 47%, 36% and 31%, respectively. The estimated 5-year OS was 73% for the CML-CP group. The results were also positive in CML-AP, CML-BP, and Ph-positive ALL. This led to the Food and Drug Administration (FDA) accelerated approval in 2012 of ponatinib 45 mg daily for patients with CML post resistance or intolerance to other TKIs, and for Ph-positive ALL (full approval in 2016 for CML-CP or in transformation, and Ph-positive ALL when no other TKI is indicated, and for T315I-mutated disease) [106, 107]. In this study, grade 3–4 hypertension was observed in 12%, any grade AOEs in 25% (severe AOEs in 20%). A subsequent report by an independent adjudication committee reported a lower frequency of adverse events (AEs) [108].

The OPTIC trial was a response-based dose-adjusted study of ponatinib in which 282 patients with CML (intolerant/resistant to ≥2 prior TKI or having the T315I mutation) were randomized to a starting dose of ponatinib 45 mg, 30 mg or 15 mg daily [65]. The dose in the first 2 arms was reduced to 15 mg daily upon achievement of CCyR (*BCR::ABL1* transcripts [IS] < 1%). The 45 mg daily arm resulted in significantly higher response rates in T315I-mutated CML compared with the 30 mg and 15 mg doses (CCyR rate 60% versus 25% versus 10%), but less so in other CML subsets (CCyR rate 54% versus 41% versus 44%). Interestingly, the 3-year OS rates were similar with the 3 dose schedules, ≈90%. The incidence of AEs, serious AEs and AOEs were significantly less than in previous studies. However the eligibility criteria addressing the cardiovascular risk status were different in the OPTIC and PACE trials, which may explain some of the differences in toxicity patterns.

In a pooled analysis of the patients on the PACE and OPTIC trials, the dose-adjusted schedules showed similar, if not better, results and significantly reduced toxicities compared with the 45 mg fixed dose (with dose reductions only for toxicities) [67]. The results with ponatinib appear to be equally promising in real-life experiences (Table 3). Breccia and colleagues reported their experience in 666 patients with CML treated with ponatinib in Italy [109]. Ninety percent of patients had prior exposure to 2 TKIs (68% to 3 and 22% to 4). Among 515 patients in CML-CP, the cumulative CCyR rate was 77%, MMR rate 65% and MR4 rate 43%. Among the 151 patients with advanced phase CML the CCyR rate was 50%, MMR rate 37% and MR4 rate 28%. With a median follow-up of 18 months, only 28/515 patients with CML-CP (5%) had died; 113 (22%) required a dose reduction from 45 mg for an AE [109].

**Table 3:** Recently reported outcomes from real-world settings with ponatinib and asciminib in second-line therapy or beyond (focusing on CML-CP data).

Study	Median follow-up/ Median time on drug	*N*	T315I	TKI line 2^nd^, 3^rd^, ≥ 4^th^	^a^MR2/ CCyR	^a^MMR	^a^≥MR4	Response based on prior ponatinib use		Survival outcome
*Ponatinib*										
^b^Heiblig et al. France [129]	26.5 mos/ 19 mos	48	25%	3,13,32	N/A	82%	N/A			3-yr OS = 80.5%
Abulafia et al. Israel [130]	14 mos/--	21	20%	5,5,11	58%	48%	29%			N/A for CML-CP cohort
Devos et al. Belgium [131]	14.9 mos/12.7 mos	33	18%	3,12,18	64%	58%	N/A			3-yr PFS = 81.6%3-yr OS = 85.3%
Sacha et al. Poland [132]	--/19.5 mos	23	26%	0,4,19	61%	48%	22%			2-yr OS = 84%
^c^Breccia et al. Italy [109]	18 mos/--	515	7%	190, 209, 116	77%	65%	43%			Median TTD = 47.2 mos
*Asciminib*				**TKI line**				**Prior Pona**	**Pona naive**	
Luna et al. Spain [115]	30 mos/11.7 mos	50	4%	88% ≥ 4^th^ line Prior Pona=38%	66%	52%	16%	CCyR= 53% MMR = 32% MR4.5 = 10%	CCyR= 74% MMR = 64% MR4.5 = 19%	Median EFS: Prior Pona= 17 mos Pona naïve= NR
^d^Khadadah et al. Russia and Canada [116]	9 mos/--	80 (CP = 65)	34%	>50% ≥ 4^th^ line Prior Pona=44%	N/A	33%	16%	MMR 17% MR4 = 6%	MMR = 47% MR4 = 24%	12-mos FFT: CML-CP Prior Pona= 28% Pona naïve= 62%
^e^Kockerols et al. Netherlands [117]	--/7 mos	49	27%	≈70% ≥ 4^th^ line Prior Pona=63%	64%	45%	32%	MR2 = 58% MMR = 39%	MR2 = 77% MMR = 56%	N/A for CML-CP cohort
Breccia et al. Italy [118]	--/8.3 mos	34	15%	74% ≥ 4^th^ line Prior Pona=59%	70%	42%	23%	N/A	N/A	N/A
Innes et al. UK [119]	--/14.4 mos	53	25%	>50% ≥ 5^th^ line Prior Pona=62%	58%	52%	NA	MMR = 48%	MMR = 65%	N/A

*MR2* molecular response log−2[BCR::ABL1 transcripts (IS) < 1%], *CCyR* complete cytogenetic response, *MMR* major molecular response [BCR::ABL1 transcripts (IS) < )0.1%], *MR4* molecular response log-4 [BCR::ABL1 transcripts (IS) < 1%], *mos* months, *N/A* not available, *CML-CP* chronic myeloid leukemia in chronic phase, *PFS* progression-free survival, *OS* overall survival, *TTD* time to drug discontinuation, *Pona* ponatinib, *EFS* event-free survival, *NR* not reached, *FFT* failure-free treatment;

^a^The responses are tabulated as the cumulative best response over the study follow-up (unless otherwise specified in the table footnote) and includes some patients who were already in this response strata before ponatinib/asciminib initiation

^b^In the study by Heiblig et al the MMR reported is the cumulative incidence by 18 months of follow-up.

^c^In the study by Breccia et al 79% of the patients with T3151 Mutation attained a response ≥MR2

^d^In the study by Khadadah et al the cumulative MMR and MR4 at 12-months are mentioned; for the CML-CP population 12- month cumulative MMR and MR4 was 40% and 19%, respectively. Responses specifically for the CML-CP population based on prior ponatinib exposure was not available.

^e^In the study by Kockerols et al the cumulative best response at 12-months are mentioned. Only 1of 10 patient with primary ponatinib failure attained an MR2 and no one achieved MMR; 2/5 patients each with secondary ponatinib failure attained a best response of MR2 and MMR, respectively; 3 of 16 and 10 of 16 with MR2 and MMR at baseline and with ponatinib intolerance maintained same response on asciminib.

All percentage figures have been rounded up

There are no head-to-head comparisons of ponatinib versus second-generation TKIs or other third-generation TKIs (such as asciminib). To provide some context regarding the potential efficacy of ponatinib versus second-generation TKIs, two studies compared the experiences in CML third-line therapy with ponatinib versus second-generation TKIs. In a systematic review by Lipton and colleagues that included 12 clinical trials comparing ponatinib to second-generation TKI in patients resistant to ≥1 prior second-generation option, ponatinib was associated with significantly superior rates of responses [96]. In another study, 354 patients with CML-CP who received third-line therapy with ponatinib on PACE-OPTIC (*n* = 150) or at MD Anderson (*n* = 31) were compared to 173 patients from MD Anderson treated with second-generation TKIs as third-line therapy (96 patients in each TKI arm after 1:1 propensity matching) [110]. Ponatinib therapy was associated with significantly higher molecular response rates. The estimated 3-year progression-free survival (PFS) rates were 83% versus 59% (*p* < 0.001) and OS rates 87% versus 83% (*p* = 0.03) for ponatinib and second-generation TKI, respectively. On multivariate analysis, ponatinib was an independent favorable factor for survival (HR 0.45; *p* = 0.003). In a combined analysis of a subset of patients from the PACE and OPTIC trials who were treated with a starting ponatinib dose of 45 mg daily and had exposure to ≥1 prior second-generation TKI, the 2-year MMR rate in second-line therapy and ≥ third-line therapy for both trials were similar at 38% and ≈30%, respectively [80]. In both trials, responses were superior in patients with T315I mutation than in those who either had no mutation or other *ABL1* KD mutations. In the OPTIC trial the 2-year PFS rate was 91% and OS rate 97% with ponatinib as second-line therapy while they were 73% and 88%, respectively, with ponatinib as ≥ third-line therapy. These figures were slightly inferior in the PACE trial [80]. Thus, ponatinib is highly active and a response-directed dose schedule leads to higher tolerability/safety and superior survival outcomes even in multi-TKI exposed or T315I-mutated CML.

### Asciminib

The next third-generation TKI to receive regulatory approval for the treatment of CML was asciminib, in 2021 [86]. Asciminib works through a novel mechanism that involves binding to the myristoyl pocket of the ABL1 and allosterically inhibiting the overactive kinase activity [111, 112].

The asciminib FDA approval was for CML-CP with previous exposure to ≥ 2 TKI or the presence of T315I mutation [86]. It was based on the ASCEMBL Phase 3 randomized trial that compared (2:1) asciminib (40 mg twice daily, *n* = 157) to bosutinib 500 mg daily (no allowance for dose-adjusted bosutinib; *n* = 76) in patients with CML-CP and prior exposure to ≥ 2 TKIs (patients were required to have had resistance to a second-line TKI or intolerance to the most recent TKI and no T315I or V299L mutation) [87]. Asciminib was used as third-line therapy in 52% patients compared with 40% for bosutinib. Prior exposure to ponatinib was noted in 15% patients on the asciminib arm and 24% patients on bosutinib. The study demonstrated significantly higher MMR rates at 6 months (the primary study endpoint) with asciminib (25% versus 12%). The long-term follow-up showed a higher MMR rate at 2 years (38% versus 16%) but the 2-year OS was similar, 97% with asciminib and 99% with bosutinib [113]. Of note, the bosutinib results in this control arm were worse than the published bosutinib data in the third-line settings. For example, Hochhaus et al. reported a cumulative 2-year MMR rate of 75% (2-year MR4 rate 60%, and MR4.5 rate 45%) in 55 patients treated with bosutinib as third-line therapy in the BYOND study [114].

The asciminib FDA approval also covered T315I-mutated CML, but the asciminib dose-schedule for this subset is 200 mg BID, which quadruples the cost (to about $1.3 million/year). This raises the issue of the treatment value of asciminib in relation to other options, e.g. allogeneic SCT. In a recent update of 48 patients with CML-CP and T315I mutations treated on the expansion cohort of the Phase 1 trial at a dose of 200 mg BID, the 2-year MMR rate was 49%. The rate of AOEs was 8% and two patients had fatal AEs [90].

In real-world experiences from Spain, Russia, Canada, the Netherlands, Italy and the United Kingdom involving > 250 patients with CML, asciminib therapy resulted in CCyR rates of 58%–70%, MMR rates of 33–52% and MR4 rates of 16–32% [115–119]. Patients with prior ponatinib exposure (mostly intolerant) had significantly lower response rates. In the study from the Netherlands, only 1/10 patients (10%) with primary ponatinib resistance achieved a CCyR with asciminib [117].

There are no trials that compare head-to-head the efficacy of ponatinib and asciminib. Table 3 shows the response rates reported with ponatinib and asciminib in real-world experiences. In prospective trials, the outcomes of patients with T315I-mutated CML appear to be better with ponatinib than with asciminib (Table 4). Also, ponatinib has shown better survival in third-line CML therapy compared with the second-generation TKIs; asciminib has not shown better survival so far in studies with shorter follow-up. Trials that compare the efficacy and toxicity profiles of ponatinib (response dose-adjusted) and asciminib in second- or ≥ third-line therapy of CML are needed.

**Table 4:** Responses with third generation TKI from prospective trials based on T315I mutation and prior lines of TKI exposure.

*Ponatinib*	Outcome measure in CML-CP	Overall	T3151 mutated	Non-T315I *ABL1* KD mutation	No *ABL1* KD mutation	1–2 prior 2G-TKI	≥3 prior 2G-TKI	Severe AOEs
**Phase I trial** [105]**, ≈1-year F/U**	**N**^a^	**43**	**12**	**15**	**13**	**40**	**-**	**81 (full cohort)**
	Cumulative CCyR	63%	75%	67%	46%	63%	–	28% at 3.5 years of follow up
	Cumulative MMR	44%	67%	53%	15%	45%	–	
**MDACC trial** [133]**, ≈ 21-months F/U**	**N**^a^	**46**	**0**	**0**	**43**	–	–	**51(full cohort)**
	CCyR at 6 months	94%	–	–	94%	–	–	Grade 3 HTN = 14% Vaso-occlusion=10%
	MMR at 6 months	83%	–	–	83%	–	–	
**PACE trial** [64]**, ≈ 5 years F/U**	**N**^a^	**257**	**64**	**67**	**136**	**100**	**157**	**270**
	MMR at 2 years	36%	59%	37%	26%	38%	31%	27%
	PFS at 2 years	68%	70%	57%	71%	72%	66%	
	OS at 2 years	85%	78%	80%	91%	82%	88%	
**OPTIC trial** [65] **(45** **mg starting dose cohort), 32 months F/U**	**N**^a^	**93**	**24**	**16**	**52**	**37**	**57**	**93**
	MMR at 2 years	33%	46%	44%	27%	38%	30%	11%
	PFS at 2 years	80%	85%	78%	78%	91%	73%	
	OS at 2 years	91%	95%	92%	89%	97%	88%	
***Asciminib***	**Outcome measure in CML-CP**	**Overall**	**T3151 mutated**	**No T3151 mutation**		**1–2 prior TKI**	**≥3 prior TKI**	**Severe AOEs**
**Phase I trial** [95]**, ≈1-year F/U**	**N**^a^	**109**	**18**	**91**		**34**	**75**	**150 (full cohort)**
	MMR at 12 months	45%	28%	48%		56%	40%	Grade 3–4 HTN = 9%
**Phase I trial expansion cohort** [90] **(asciminib** = **200** **mg/day)**						**Prior ponatinib**	**Ponatinib naive**	
	**N**^a^	**45**	**45**	**0**		**26**	**19**	**45**
	MMR at week 96	49%	49%	–		35%	68%	8%
**ASCEMBL trial** [87],≈ **15 months F/U**						**1–2 prior TKI**	**≥3 prior TKI**	
	**N**^a^	**157**	**0**	**157**		**82**	**75**	**156**
	Cumulative 24-week MMR	25%	–	25%		29%	21%	3% (2 deaths due to AOE)
***Olverembatinib***	**Outcome measure in CML-CP**	**Overall**	**T3151 mutation**	**Non-T315I** ***ABL1*** **KD mutation**	**No*ABL1*** **KD mutation**	–	**–**	**Severe AOEs**
**Phase I/II trial** [134]**, ≈ 3-years F/U**	**N**^a^	**126**	**90**	**9**	**22**	**–**	**–**	**127**
	Cumulative MMR	56%	≈ 67%^3^	≈ 55%^3^	9%	–	–	5% Grade 3–4 CVE = 12%

CML-CP chronic myeloid leukemia in chronic phase, *ABL1* KD *ABLI* kinase domain, 2 G second generation, TKI tyrosine kinase inhibitor, AOE arterio-occlusive events, F/U follow-up, CCyR complete cytogenetic response, MMR major molecular response, MDACC MD Anderson Cancer Center Houston, HTN hypertension, PFS progression-free survival, OS overall survival, CVE cardio-vascular events

^a^“N” depicts the denominator number of patients for the corresponding column amongst whom the outcome measures or AOEs have been reported in the rows below them.

### Olverembatinib

Olverembatinib (HQP1351) is a new third-generation TKI that showed in cell lines the potential capability to inhibit both wild type *BCR::ABL1* and T315I-mutated *BCR::ABL1* [120]. The drug is approved in China for the treatment of adults with TKI-resistant CML in CP or AP harboring the T315I mutation. Because of the approval in China (1.4 billion people), and because of the encouraging results, detailing the reported results is valuable.

In pre-clinical studies, olverembatinib has shown an exciting efficacy profile across CML mutants, compared with second- and third generation TKIs **(**Table 2**)**. In a study of 101 patients with CML (CML-CP = 86, CML-AP = 15) treated with olverembatinib, 62% had T315I mutation and 83% had ≥2 TKIs. At the 5-year follow-up, in CML-CP, the CCyR rate was 71%, the MMR rate was 55%, and the estimated 4-year PFS rate 85.6% [121]. Grade 3/4 adverse cardiovascular events were noted in 12% of patients, most commonly hypertension. One patient each had a retinal vein occlusion, CNS infarction, and a myocardial infarction. In CML-AP, the CCyR rate was 40%, the MMR rate 40%, and the estimated 4-year PFS rate 50%. In another update of 64 patients with CML-CP (*n* = 41) or CML-AP (*n* = 23) and T315I mutation, the results were encouraging. In CML-CP the CCyR rate was 71%, the MMR rate 58%, the estimated 3-year PFS rate was 86% and OS rate 95% [122]. In CML-AP, the CCyR rate was 52%, the MMR rate 48%, the estimated 3-year PFS rate was 57% and OS rate 70%. A recent US study treated 30 patients with olverembatinib 30, 40 or 50 mg every other day (24/30 with ≥3 prior TKIs; prior ponatinib 21/30; ponatinib resistance 17/30). The CCyR rate in CML-CP was 69% and the MMR rate 44%. Among patients with ponatinib resistance 5/9 (56%) achieved CCyR, and 6/11 (55%) achieved MMR [123].

Table 4 shows the efficacy of the third-generation TKIs from prospective clinical trials, stratified based on T315I mutation data and the line of TKI salvage.

## Addressing the most frequent question in CML management: changing TKI therapy in a patient with *BCR::ABL1* transcripts (IS) < 1% but not in MMR, DMR or undetectable levels

The absence of MMR by one year of TKI therapy is considered a “warning” in the ELN recommendations. The true clinical risk of not achieving this endpoint may be over-estimated. Thus, in patients who do not have high-risk CML features (high-risk additional cytogenetic abnormality, mutations in genes such as *ASXL1*) and in whom TFR is not an aim, it is reasonable to continue the same TKI at the same dose, provided the patient tolerates the drug well, maintains compliance to therapy and is monitored every 3–6 months. As detailed earlier, in patients with persistent low-level *BCR::ABL1* transcripts (IS) 0.1–1%, the long-term CML-specific survival is excellent (10-year OS rate about 90%). Changing to a third-generation TKI in such situations may increase the toxicities and cost, without improving the long-term outcome.

## Approach to patients with T315I mutation

The treatment options in patients with *ABL1-*T315I mutation have evolved with the approval of ponatinib and asciminib, and the promising results from reported and ongoing trials of olverembatinib. Cross-trial comparison of data shows that ponatinib may result in better responses compared with asciminib in T315I-mutated CML and should be the preferred option in the absence of absolute contraindications. Also, as discussed earlier, the approved dose of asciminib for T315I-mutated CML has a prohibitive cost (about $1.3 million/year) and becomes a major “financial toxicity,” given that these drugs need to be continued for years.

Options beyond TKIs should be explored. In the early trials with omacetaxine mepesuccinate, the drug led to a steady reduction in T315I-mutated *BCR::ABL1* transcripts, rendering these patients amenable to treatment with earlier-generation TKIs [124]. Small patient series have also reported similar activity with IFN alpha sequenced or combined with TKIs [125–127]. With the advent of TKIs that possess activity against T315I-mutated CML, these options are rarely used, but remain relevant in the rare patients who are unable to tolerate third-generation TKIs or who cannot access or afford them. Allogeneic SCT remains a one-time appropriate option in T315I-mutated CML, but preferably after a trial of therapy with ponatinib, as retrospective data have shown the superiority of ponatinib over SCT in CML-CP with T315I mutation [128].

## Conclusion

As patients with CML have a near-normal life span on TKI therapies, it has become increasingly important to clarify the goals of therapy (survival; TFR) and the likelihood that such goals can be achieved on different TKIs, and then to revisit the treatment milestones that have been standard for the past 2 decades. It is also important to clarify the benefit versus toxicity (clinical and financial) of changing TKIs more frequently than necessary in pursuit of goals that may not be achievable (for example, changing TKIs in a patient with detectable *BCR::ABL1* [IS] transcripts > 0.01 % or > 0.1% after >5 years of TKI therapy in pursuit of TFR). In patients who are not candidates for TFR, any response below *BCR::ABL1* (IS) transcripts <1% is a reasonable goal. More stringent molecular goals could be considered in patients in whom a TFR is an aim. In patients with non-prohibitive TKI toxicity, dose reductions should be the first step before a TKI change since dose reductions in the right context are effective and safer, often leading to better treatment compliance. This review attempts to analyze these issues and stimulate discussions as to the most appropriate courses of action in the management of frontline and later lines of TKI therapies in CML in the community practice.
